# Comparison of sampling methods for the determination of volatile organic compounds in consumer aerosol sprays

**DOI:** 10.1038/s41598-023-41911-x

**Published:** 2023-09-13

**Authors:** Sungyo Jung, Myoungho Lee, Geonho Do, Boowook kim, Kiyoung Lee, Kyung-Duk Zoh, Chungsik Yoon

**Affiliations:** 1 Department of Environmental Health Sciences, Graduate School of Public Health, Seoul National University, Seoul, Republic of Korea; 2 Institute of Health and Environment, Seoul National University, Seoul, Republic of Korea

**Keywords:** Risk factors, Public health

## Abstract

Many studies have evaluated the hazardous substances contained in various household chemical products. However, for aerosol spray products there is currently no international standard sampling method for use in a component analysis. The aim of this study was to develop an appropriate sampling method for the analysis of volatile organic compounds (VOCs) in consumer aerosol sprays. Two different sampling methods, spraying (into a vial) and perforating (and transferring the contents into a vial), were used to evaluate the levels of 16 VOC components in eight different aerosol spray products. All eight products contained trace amounts of hazardous VOCs, and a quantitative analysis showed that, for the same product, VOC concentrations were higher when spraying than when perforating. Using the spraying method, average toluene, ethylbenzene, p-xylene, o-xylene, and styrene concentrations were 1.80-, 2.10- 2.25-, 2.03-fold, and 1.28-fold higher, respectively, than when using the perforating method. The spraying method may provide more realistic estimates of the user's exposure to harmful substances and the associated health risks when using spray products. Of the two representative methods widely used to analyze harmful substances in consumer aerosol sprays, the spraying method is recommended over the perforating method for the analysis of VOCs.

## Introduction

Consumer spray products are chemical products commonly used in the household environment^1^. During the COVID-19 pandemic, the frequency and amount of consumer spray product use (e.g., disinfectants, sanitizers, and detergents) in indoor environments increased worldwide^2^. However, there is growing concern that the hazardous substances these products contain could adversely affect human health^1,3–6^. Several studies have analyzed the hazardous substances in various consumer spray products, such as detergents, coatings, diffusers, and disinfectants^7–13^. Building on these studies, further assessments of emission rates and consumer exposure to hazardous substances in the indoor environment associated with the use of certain products have been conducted^14–20^.

Previous studies have analyzed volatile organic compounds (VOCs) in various household chemical products, but there is no internationally standardized sampling method available for aerosol spray products. There are two options for sampling the contents of a consumer aerosol sprays: (1) spraying the spray into the vial or (2) perforating the spray and transferring a sample into a vial by syringe. During spraying, the internal chemical components of the spray can become mixed due to various factors such as the internal pressure of the gas, drag forces within the nozzle that are induced by the material and shape of the nozzle, and fluid turbulence^21–23^. Therefore, the contents collected through spray sampling may not match the original composition of the spray. Alternatively, when sampling the contents of an aerosol spray by perforating, there is a risk of contamination during the sampling process. Additionally, substances with high volatility can rapidly evaporate into the air, leading to undervaluation of their concentrations. Moreover, the most critical issue in terms of safety is that puncturing the spray presents an explosion risk due to the internal gas pressure. Sample collection from aerosol sprays can therefore be dangerous depending on the collection method. The Ministry of Environment of the Republic of Korea therefore recommends the spraying method when analyzing the contents of consumer aerosol sprays for determining VOCs^24^. One previous study sampled an aerosol spray by puncturing the spray and then analyzing the contents for determining aerosols^25^. However, few studies have attempted to collect such samples and there is no international standard sampling method for component analysis of consumer aerosol sprays.

This study compared the two sampling methods used for the analysis of the contents in consumer aerosol spray products containing hazardous substances and proposed an appropriate sampling method for determining VOCs in the spray.

## Materials and methods

### Study materials

Eight consumer aerosol sprays were selected based on the following three criteria.

1. Products were registered in the Consumer Environmental Safety Information System (https://ecolife.me.go.kr/ecolife/), which is an open information portal where consumers can access information on chemical products. The portal was developed, and is operated, by the South Korean Ministry of Environment^24^.
2. Products were pressurized with gases such as liquefied petroleum gas or dimethyl ether and applied by spraying.
3. Products were currently on sale in Korea and ranked in the top 30 products in terms of the number of positive purchase reviews on the seller's website.

Four cleaning products, two deodorants, and two coating products from seven manufacturers were selected based on these criteria.

### Sampling methods

**Figure 1:**
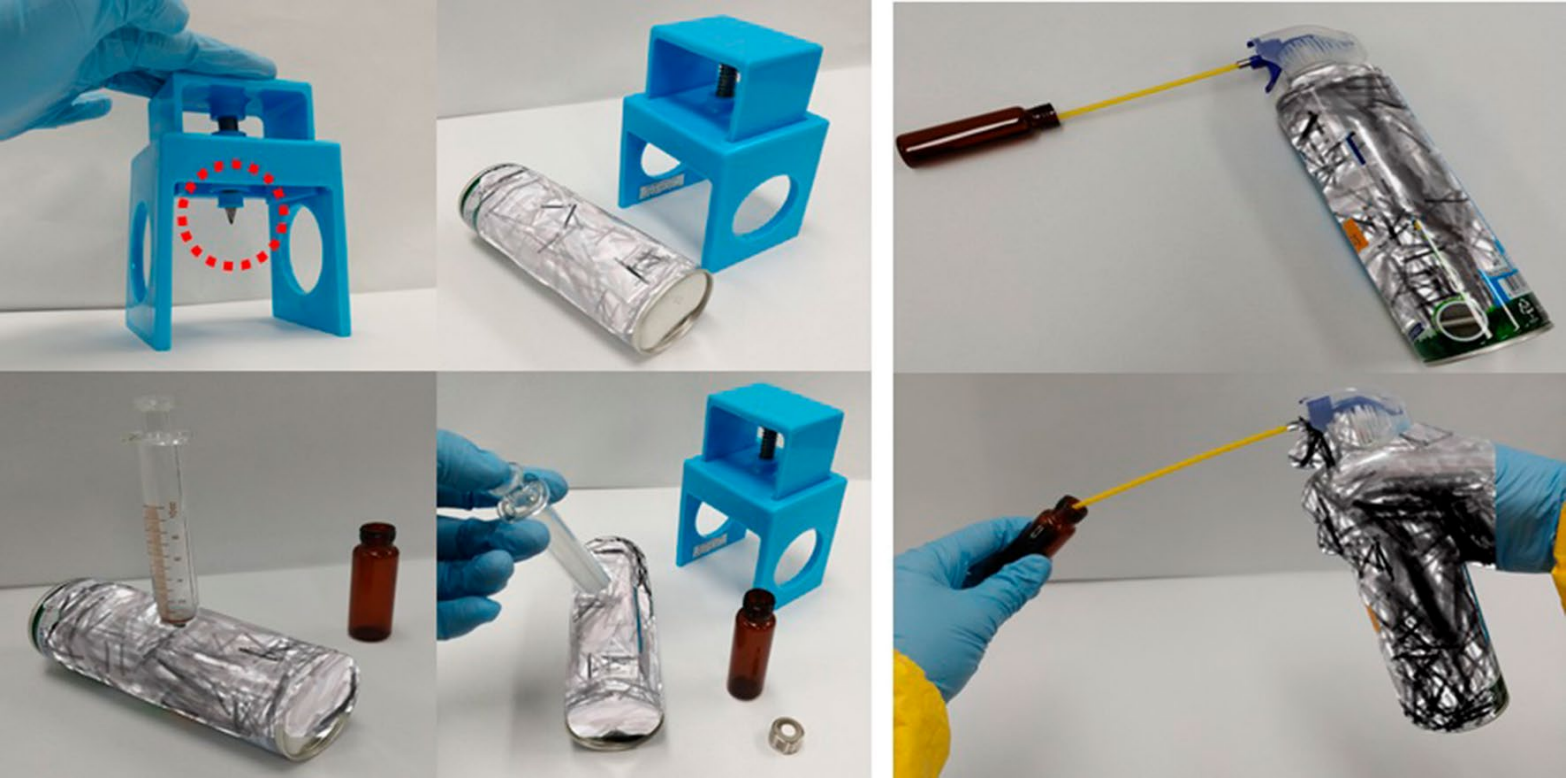
Procedures used to sample the components of consumer aerosol spray products (Left: the perforating method, Right: the spraying method).

Two sampling methods were used to collect the contents of the consumer aerosol sprays in this study. In the first method (i.e., spraying), the spray products were sprayed into a clean 20-mL vial with a spray nozzle, as recommended by the South Korean Ministry of Environment's regulations on “the safety management of consumer chemical products and biocides”^24^. In the second method (i.e., perforating), which had been applied in a previous study^25^, the consumer aerosol spray was punctured with a mechanical device after the internal pressure was reduced by expelling the contents until less than one-third remained, followed by refrigeration for > 24 h. When puncturing the aerosol spray, it was placed inside a clean bench with a laminar flow hood and appropriate personal protective equipment was worn for safety reasons. The contents of the punctured product were then transferred to a clean 20-mL vial using a glass syringe. The vials containing the samples obtained by the two methods were sealed and stabilized by refrigerating for at least 30 min, and then 0.2 g of sample was placed in fresh 20 mL brown vials to prepare the final samples for analysis. Before aliquoting the final sample, all stabilized samples were mixed well by vigorous shaking. For all aerosol sprays, the content of the sprays was reduced to less than one-third remined, and they were stored in refrigeration for over 24 h before sampling to set up a similar sampling condition. Additionally, all vials used in this sampling process were washed and stored in refrigeration for over 24 h, just like the aerosol spray products, before being used for sampling. To compare the results via a statistical analysis, at least three samples of each product obtained using the same method were prepared and analyzed. The sampling procedure for each sampling method is shown in Fig. 1.

### Quantitative analysis

We referred to the information provided on the label of each product to comprehensively evaluate the various hazardous VOCs that consumers may be exposed to. A total of 57 VOC components certified in accordance with ISO 17,034 and ISO/IEC 17,025 were initially analyzed using certified reference material (CRM)-grade standard materials diluted by methanol (ISO 17,943 57 Component VOC Mix Supelco—44,926-U [Supelco, Bellefonte, PA, USA]). The final analysis was conducted on the 16 VOCs detected in at least one samples.

The quantitative analysis of VOC concentrations in samples was conducted by gas chromatography-mass spectrometry (GC–MS; 7890A-5975C; Agilent, Santa Clara, CA, USA) using headspace injection. A DB-5 MS UI column (Agilent; length, 60 m; inner diameter, 0.25 mm; film thickness, 0.25 μm) was used for the analysis. A headspace syringe with a volume of 2.5 ml and scale length of 60 mm (MSH 02-00B; CTC Analytics, Zwingen, Switzerland) was used in the analysis. Samples were agitated for 20 min at 500 rpm and 90 °C. After agitation, 50 μl injection of the headspace gas was injected into the GC–MS at a rate of 1000 μl/s, with the headspace syringe heated to 95 °C by an autosampler. One laboratory blank was included for every 10 samples to assess instrumental drift and performance. Standard solutions of the 16 VOCs were prepared using methanol as a solvent. The specific headspace GC–MS analysis conditions were established considering environmental guidelines and previous studies^9,24,26–29^ and are given in Table S1.

**Figure 2:**
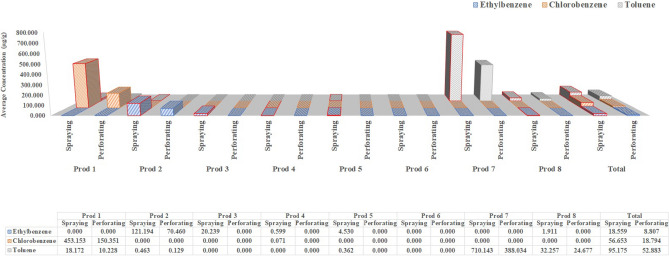
Concentrations of high-concentration group substances in different spray products using two sampling methods (perforating and spraying).

**Figure 3:**
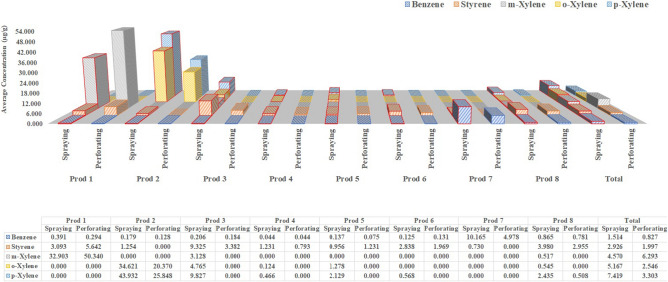
Concentrations of medium-concentration group substances in different spray products using two sampling methods (perforating and spraying).

**Figure 4:**
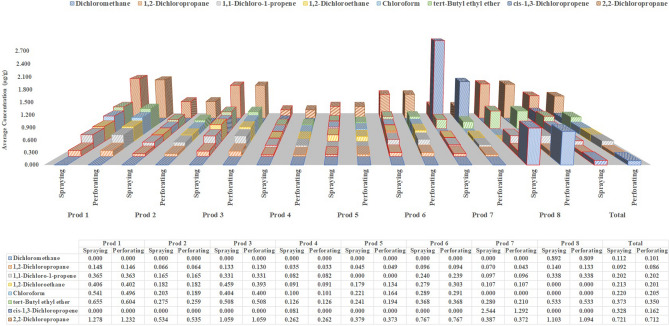
Concentrations of low-concentration group substances in different spray products using two sampling methods (perforating and spraying).

To clearly visualize the analysis results, 16 VOCs were classified into three groups, i.e., high-, medium-, and low-concentration groups, based on the level of concentration shown in Figs. 2, 3 and 4, respectively. The limit of detection (LOD) of each VOC and not detected (ND) rate are presented in Table S2^30^.

### Statistical analysis

Statistical tests were conducted to identify significant differences between mean values (parametric) or median values (nonparametric) for the two sampling methods. Each test group had at least three samples for each sampling method and product.

For the groups that exhibited normally distributed data according to the Shapiro–Wilk test, two-sample t-tests were performed to determine whether two groups had statistically significantly different mean values after Levene's test to assess homogeneity of variance between groups. For groups that did not exhibit normally distributed data, the Wilcoxon rank sum test was used to determine whether two groups had statistically significantly different median values. In all statistical analyses, p < 0.05 was taken to indicate statistical significance. P-values of each statistical analysis using quantitative analysis of the 16 VOCs in consumer aerosol sprays are shown in Table S3.

### Evaluation of concordance

To compare the VOC concentrations between the two sampling methods, we used the four indicators listed below. For consistency, we calculated the relative percentage difference in the maximum mean change^31^.

**Figure 5:**
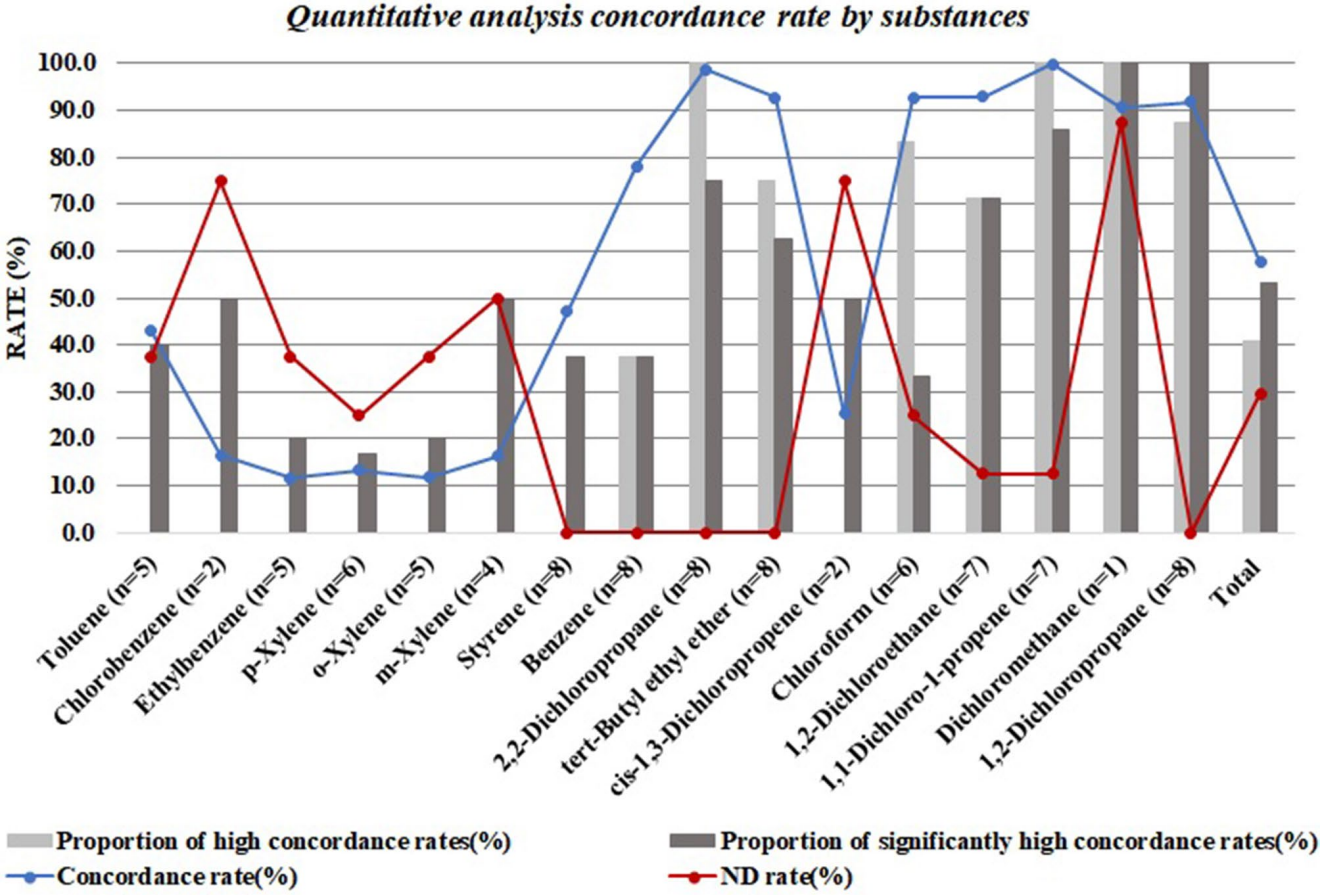
Concordance rate between sampling methods by substance. Light grey bars: the proportion of consumer products with a concordance rate between sampling methods > 90% for the same VOC. Dark grey bars: the proportion of consumer products for which there was no statistically significant difference (p < 0.05) between sampling methods for the same VOC.

1. Concordance rate (%).
  : [1 – {|Spraying—Perforating|/Max(Spraying, Perforating)}] × 100 (%).
  This indicator, i.e., the difference between the largest and smallest values obtained using the two sampling methods, is expressed as a percentage by dividing by the larger of the two values. The closer this value is to 100%, the more consistent the values obtained using the two sampling methods are.
2. ND rate (%).
  ① {(Number of VOCs ND)/(16)} × 100 (%)
    This indicator is the proportion of VOCs ND for each spray product (Fig. S1).
  ② {(Number of spray products in which a specific VOC was ND)/(8)} × 100 (%).
    This indicator is the proportion of spray products in which each VOC was ND (Fig. 5).
3. Proportion of high concordance rates (%).
  ① {(Number of VOCs with a concordance rate > 90%)/(16)} × 100 (%).
    This indicator is the proportion of VOCs with a concordance rate > 90% between the two sampling methods for each spray product (Fig. S1). The higher the value, the more consistent the 16 VOC concentrations of spray products are between the two sampling methods.
  ② {(Number of spray products with a concordance rate > 90%)/(8)} × 100 (%).
    This indicator is the proportion of spray products with a concordance rate > 90% between the two sampling methods for each VOC (Fig. 5). The higher the values, the more consistent the contents of the eight spray products are between the two sampling methods for each VOC.
4. Proportion of significantly high concordance rates (%).
  ① {(Number of VOCs with no statistically significant differences)/(16)} × 100 (%).
    This indicator is the proportion of VOCs in each spray product for which there are no statistically significant differences (p < 0.05) between the two sampling methods (Fig. S1). The higher the value, the greater the concordance rate between the two sampling methods.
  ② {(Number of spray products with no statistically significant difference) / (8)} × 100 (%).
    This indicator is the proportion of spray products for which there are no statistically significant differences (p < 0.05) between the two sampling methods for each VOC (Fig. 5). The higher the value, the greater the concordance rate between each VOC.

When calculating (4), the number of groups with statistically significant differences at the p < 0.05 level was determined through statistical testing, i.e., a two-sample t-test or the Wilcoxon rank sum test. Then, the proportion of groups with no statistically significant differences was calculated to estimate the concordance rate.

### Comparison of reproducibility

In order to compare the reliability of the two sampling methods used in this study, reproducibility was assessed. Different samples collected from each aerosol spray using the two sampling methods were analyzed under the same experimental conditions. Therefore, to evaluate reproducibility, the standard deviation (SD) and relative standard deviation (RSD) were calculated for each of the 16 analyzed substances for each aerosol spray and for each sampling method. A lower value of SD and RSD indicates better reproducibility for the corresponding sampling method. The RSD (%) was calculated as the percentage of the standard deviation relative to the mean. By analyzing and comparing the SD and RSD values for each substance and aerosol spray under different sampling methods, the reproducibility of each sampling approach was assessed.

## Results

### Composition of consumer aerosol sprays

The chemical compositions of each spray product, which were confirmed by reference to the Consumer Environmental Safety Information System and product labels, are shown in Table S4. Of the eight products evaluated, there were four cleaning products, two coating products, and two deodorants.

We have listed the substances with existing Cas No. among the components provided in the product label in Table S4. Since specific content information for each component was not provided, we were unable to evaluate the risk or toxicity. However, we attempted to classify them into IARC classifications based on the carcinogenicity monographs provided by the International Agency for Research on Cancer (IARC). Ethanol, which is classified as a Group 1 substance by the IARC, was found in two cleaning products and two deodorant products, and cocamide diethanolamine, which is classified as an IARC Group 2B substance, was found in one cleaning product^32^.

The concentrations of these substances were not provided in the product labels. In some cases, information about the raw materials used in the manufacturing process was listed rather than the actual active substance, making it difficult to evaluate the chemical characteristics, toxicity, and health hazards of the spray products.

### Chemical properties of the analyzed components

The chemical properties of the 16 VOCs analyzed in this study are shown in Table S5. The table includes information obtained from PubChem, operated by the National Library of Medicine. The heat of vaporization was estimated by the Clausius–Clapeyron equation based on the chemical properties provided by PubChem^33^.

In previous studies, substances with a high vapor pressure were found to be easily vaporized due to their high volatility under the same temperature and pressure conditions^34,35^. Additionally, substances with a low boiling point and heat of vaporization have high volatility and are easily vaporized^35^. According to Fick's law, substances present in high molar concentrations tend to diffuse easily into the surroundings due to their high diffusivity^36^.

### Quantitative analysis

The concentrations of the 16 VOCs according to sampling method are shown in Figs. 2, 3 and 4. Three substances were present in high concentrations (mean, 8.8 − 95.2 μg/g; range: 8.8 − 700 μg/g) and were classified into the high-concentration group; five substances were present in moderate concentrations (mean, 0.8 − 7.4 μg/g; range: 0.8 − 50 μg/g) and were classified into the medium-concentration group; and eight substances were present in low concentrations (mean, 0.1 − 0.7 μg/g; range: 0.1 − 2.5 μg/g) and were classified into the low-concentration group. Figure 2 shows the concentrations of toluene, chlorobenzene, and ethylbenzene concentrations, all of which were classified into the high-concentration group (ND rate, 37.5%, 75.0%, and 37.5%, respectively). Toluene and ethylbenzene were detected in five of the eight consumer aerosol sprays at concentrations of 0.13 − 710.14 and 0.60 − 121.19 μg/g, respectively. Chlorobenzene was only detected in two of the eight products, with an average concentration of 0.07 μg/g in one sprayed product, and 453.2 and 150.4 μg/g in the other sprayed and perforated product, respectively. The results for the high-concentration group revealed higher concentrations of products with spraying compared to perforating.

Figure 3 shows the concentrations of p-xylene, o-xylene, m-xylene, styrene, and benzene, all of which were in the medium-concentration group (ND rate: 25%, 37.5%, 62.5%, 0%, and 0%, respectively). Styrene and benzene were detected in all eight consumer aerosol sprays, with concentrations of 0.73 – 9.33 and 0.04 – 10.17 μg/g, respectively. p-xylene and o-xylene were detected at levels of 0.47 – 43.93 and 0.12 – 34.62 μg/g in six and five consumer aerosol sprays, respectively, but were detected in only four and three products, respectively, when using the spraying method. m-xylene was detected in only three products, at concentrations of 0.52 – 50.34 μg/g. In one product the concentration obtained using the perforating method (50.34 ± 9.09 μg/g) was higher than that obtained using the spraying method (32.90 ± 11.40 μg/g), but in the other two products it was only detected by the spraying method.

Figure 4 shows the concentrations of the eight substances in the low-concentration group. Dichloromethane and cis-1,3-dichloropropene were detected in only two and one consumer aerosol sprays, respectively. Similar to the high- and medium-concentration groups, the concentrations of eight low-concentration group VOCs were also higher in spraying method than perforating method samples of the same product. The concentrations of the 16 VOCs in each consumer aerosol spray obtained using both sampling methods are presented in Table S2.

### Concordance rate evaluation

Figure S1 and Table S6 show the concordance rates in VOC concentrations for all product samples between the two methods. The concordance rate between the VOC sampling methods was in the range of 50.3 – 83.8% (average, 65.6 ± 11.7%), with an ND rate range of 12.5 – 43.8% (average, 28.9 ± 10.5%). Among the eight consumer aerosol sprays, the proportion of VOCs for which the concordance rate between the two sampling methods was above 90% was in the range of 18.2 − 77.8% (average, 46.4 ± 17.0%). The proportion of VOCs for which there was no statistically significant difference (p < 0.05) between sampling methods for the same spray product was in the range of 18.2 – 75.0% (average, 52.5 ± 17.9%). Therefore, concentrations of chemical constituents of the same consumer aerosol spray may differ by an average of 34.4% depending on the sampling method.

Figure 5 and Table S7 show the concordance rates between the sampling methods for each VOC. The average concordance rate between the sampling methods for the 16 VOCs was 57.7 ± 37.2% (range: 11.6 – 99.8%), with an average ND rate of 29.7 ± 29.5% (range: 0.0 – 87.5%). Additionally, the proportion of consumer aerosol sprays with a concordance rate between the two sampling methods > 90% was in the range of 18.2 – 77.8% among the 16 VOCs (average, 46.4 ± 17.0%). The proportion of consumer aerosol sprays for which there was no statistically significant difference (p < 0.05) in the concentration of a given VOC (p < 0.05) between the two sampling methods was in the range of 16.7 – 100.0% (average, 53.1 ± 27.0%).

As shown in Fig. 5, there were no consumer aerosol sprays for which the concordance rate between sampling methods was > 90% among all high-concentration group substances (toluene, chlorobenzene, and ethylbenzene), although a concordance rate > 90% was seen for four of the medium-concentration group substances (p-xylene, o-xylene, m-xylene, and styrene). Furthermore, for five VOCs (chlorobenzene, ethylbenzene, p-xylene, o-xylene, and m-xylene), the concordance rate between sampling methods was < 17%. For toluene and styrene, the concordance rates between sampling methods were 43.1 ± 29.6 and 47.1 ± 31.8, respectively. There were no consumer aerosol sprays for which the concordance rates between sampling methods were > 90% for cis-1,3-dichloropropene, which was detected in only two products. Among all products, the average concordance rate between sampling methods was 25.4 ± 35.9%.

### Comparison of reproducibility

**Figure 6:**
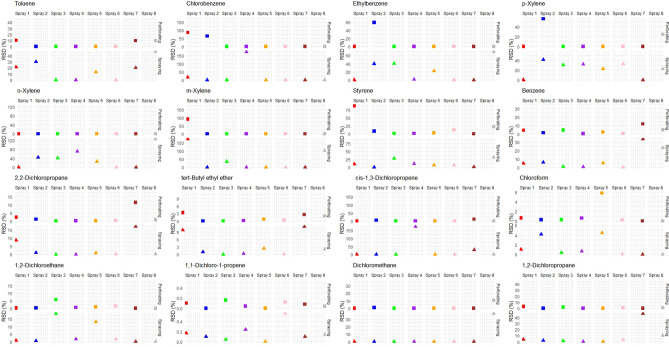
Distribution of RSD values in quantitative analysis of eight consumer aerosol sprays for each substance.

The standard deviations (SD) and relative standard deviations (RSD) for each analyzed substance in each aerosol spray under different sampling methods are presented in Table S2. Additionally, the average RSD values and detection rates for each substance among all aerosol spray products are shown in Table S8. The individual RSD values for the eight sprays according to sampling methods for each substance are shown in Fig. 6, and Fig. S2 presents boxplots based on the RSD values of the eight sprays for each substance and sampling method, allowing for an overview of the values for each group.

In the analysis of 8 aerosol spray products, 6 substances (Toluene, Chlorobenzene, Ethylbenzene, p-Xylene, o-Xylene, m-Xylene) detected at relatively higher concentrations exhibited RSD values of 30% or higher, indicating higher variation compared to other substances. Additionally, among the 16 substances, 9 substances (Chlorobenzene, o-Xylene, m-Xylene, Benzene, 2,2-Dichloropropane, tert-Butyl ethyl ether, cis-1,3-Dichloropropene, 1,2-Dichloroethane, Dichloromethane, 1,2-Dichloropropane) showed higher RSD values in the spraying sampling method compared to the perforating sampling method, resulting in larger variations.

## Discussion

Household chemical products contain many substances that are potentially hazardous to health. However, there is no international standard method for sampling the components of aerosol spray products for use in health risk evaluations. Therefore, we compared two frequently used sampling methods in a quantitative analysis of 16 hazardous VOCs in consumer aerosol sprays.

Sixteen hazardous VOC components had concentrations above the LOD among consumer aerosol spray products, of which seven were potentially carcinogenic substances classified by the IARC carcinogenicity classification: benzene, 1,2-dichloropropane, styrene, dichloromethane, ethylbenzene, chloroform, and dichloroethane. According to component data for provided by the Consumer Environmental Safety Information System and product labels, the consumer aerosol sprays analyzed in this study contained hazardous substances such as ethanol and cocamide diethanolamine. However, they were not quantified in this study because they are not among the standard materials (Supelco—44926-U ISO 17943 57 Component VOC Mix).

We considered the possibility that the 8 aerosol spray products used in this study might also contain other hazardous chemical substances, in addition to those listed on their labels. Consequently, in this study, we used the standard material containing 57 different VOC compounds to monitor as many diverse VOC substances detectable in spray products as possible. However, the standard material used in this study inherently contained methanol as a basic solvent, so we diluted it with methanol to prepare standard solutions. Nonetheless, due to the unknown composition and concentrations of substances in the aerosol sprays used in this study, we refrained from adding methanol or any other solvent during the analysis. Additionally, in the preliminary test before quantitative analysis, carbon disulfide and methanol were used as solvents. However, it was observed that many spray products showed phase separation in two layer or remained undissolved, resulting in aggregation within the solvents. The concern was that the addition of such solvents might cause unpredictable effects on the sample components or affect the homogeneity of sample, leading to different analytical results from the actual spray components.

For the 16 VOCs, spraying method resulted in higher concentrations for all eight products tested than the perforating method. During the perforation process, substances with high volatility are easily vaporized. Additionally, substances present at high concentrations were underestimated due to their high diffusivity, due to which they easily diffuse into the surrounding environment. There may also be a loss of VOCs during spray sampling but it was still confirmed to be a better option than perforating.

Regarding reproducibility, 9 substances showed higher RSD values in the spraying sampling method compared to the perforating sampling method among 16 substances. However, for 4 substances (Benzene, 2,2-Dichloropropane, tert-Butyl ethyl ether, 1,2-Dichloroethane), RSD values were below 10% for both sampling methods, with slight differences between the two methods. While there were significant differences in RSD values among sprays for the same substances, the RSD values were excessively high in some substances. In a previous study conducted to analyze the components of aerosol spray, five substances were analyzed by GC–MS with headspace injection using solvents such as ethyl lactate (EL), DMSO, and DMF^37^. When EL was used as the solvent, it presented high recovery and reproducibility for five substances. In this study, Benzene and 1,2-Dichloroethane were common with the previous study, also showed low RSD values. However, the results from the previous study showed higher consistency. Therefore, when information about the spray components and considerations regarding solubility is available, we recommend conducting the component analysis using EL as the solvent to enhance stability during analysis.

Some substances showed high RSD values during spraying sampling, indicating that content composition may not uniform during spraying. Additionally, some substances showed significant variations in RSD values among the sprays for the same substance. During spraying, different variables such as gas pressure and nozzle shape may affect the composition of the aerosol sprayed^21–23^. When using spraying sampling method, these characteristics and variables of each spray may be reflected in the sampling process, potentially resembling the composition of the aerosol to which generated during spray usage. To obtain more precise data, it would be necessary to increase the sample size under various conditions for the same spray product. However, to accurately assess these variables, it is necessary to conduct exposure assessments of users after actual spray usage. In cases where risk assessments are conducted without data obtained from actual exposure assessments, it could be advisable to use component analysis data obtained through the spraying sampling method. In such instances, although there may be relatively high variability in the analysis data results, it could reflect the inter-spray variability due to differences in the composition of the content being sprayed.

In this study, we used IARC carcinogenicity classification as a supplementary indicator to argue that the concentration of certain potentially carcinogenic substances may be underestimated when perforating sampling is conducted. Since risk assessment was not conducted in this study, there is a limitation to estimating the health risks associated with user exposure during actual spray usage based solely on the carcinogenicity of detected substances and analytical results from the product.

## Conclusions

We recommend the spraying method for analyzing consumer aerosol spray components and, by extension, health hazards for the following three reasons.

First, the spraying method is likely to obtain higher concentrations than the perforating method, which underestimated VOC concentrations in this study. Evaluation of the worst-case scenario with respect to human health is necessary when analyzing the VOC concentrations to which users may be exposed. Second, volatile substances that easily evaporate or have high diffusivity will readily diffuse into the surrounding environment, and are more likely to be underestimated when sampling by the perforating method. Finally, when using spray sampling method, characteristics and variables of each spray may be reflected in the sampling process. It could reflect the differences in the composition of the aerosol generated during spray usage and the inter-spray variability into the analysis process.

### Supplementary Information

Supplementary Information.

## Data Availability

All data that support the findings of this study are shown from Tables S1–S8 in the Supplementary Information (see Electronic Supplementary Material). The more detailed datasets of the current study are available from the corresponding author on reasonable request.
