# Supplementary material for: Comparison of sampling methods for the determination of volatile organic compounds in consumer aerosol sprays
Source: Sci Rep. 2023 Sep 13;13:15143. doi: 10.1038/s41598-023-41911-x (PMC10499907; doi:10.1038/s41598-023-41911-x)
Supplement: Supplementary file 1 — Supplementary Information. [file 41598_2023_41911_MOESM1_ESM.docx]

**Supporting Information (SI)**

The analytical conditions for the quantitative analysis of consumer aerosol sprays by headspace GC-MS are presented in Table S1. The concentrations of the 16 VOCs in the spray products determined using the two sampling methods are shown in Table S2. The p-values for the quantitative analyses of the 16 VOCs are shown in Table S3. The product information and chemical components for each consumer aerosol spray used in the study are presented in Table S4. The chemical properties and detection rates of the analyzed VOCs are presented in Table S5. The concordance rates between sampling methods by product are presented in Figure S1 and Tables S6. The concordance rates between sampling methods by chemical substance are presented in Table S7. Relative standard deviation values of substances among analyzed eight consumer aerosol sprays are presented in Table S8. Distribution of relative standard deviation values among 8 spray products for each substance are presented in Figure S2.

**Table of Contents**

**Table S1.** Analytical conditions for the quantitative analysis of consumer aerosol spray product by headspace gas chromatography-mass spectrometry.

**Table S2.** Quantitative analysis of 16 VOCs in spray products using two sampling methods.

**Table S3.** P-values obtained by statistically comparing the two sampling methods applied to 16 VOCs in consumer aerosol spray products.

**Figure S1.** Concordance rate between sampling methods by spray product.

**Table S4.** Product information and chemical components for each consumer aerosol spray product used in the study.

**Table S5.** Chemical properties and detection rates of the VOCs analyzed in the study.

**Table S6.** The concordance rates between sampling methods by product.

**Table S7.** Quantitative analysis concordance rate according to sampling method by substances.

**Table S8.** Relative standard deviation (RSD) of substances among analyzed eight consumer aerosol sprays.

**Figure S2.** Distribution of RSD values in quantitative analysis of eight consumer aerosol sprays for each substance.

**Table S1.** Analytical conditions for the quantitative analysis of consumer aerosol spray product by headspace gas chromatography-mass spectrometry.

| **Parameter** | **Analytical Conditions** | | |
| --- | --- | --- | --- |
| Instrument | Gas chromatography-mass spectrometry  (7890A-5975C; Agilent, USA) | | |
| Injector | Autosampler  (PAL COMBI-x; BGB Analytik, Switzerland) | | |
| Head-space injection |  | | |
| Pre-heating/agitation temperature | 90°C | | |
| Pre-heating/agitation time | 25 min | | |
| Inlet temperature | 230°C | | |
| Injector volume | 50 µl | | |
| Split ratio | 10:1 | | |
| Column | DB-5MS UI  (60 m × 0.25 mm × 0.25 µm; Agilent) | | |
| Carrier gas | Helium (He), 1.0 ml/min | | |
| Column temperature |  | | |
| Oven temperature (hold time) | 40°C (2.0 min)→60°C | 60°C (2.0 min)→120°C | 120°C (1.0 min)→230°C |
| Heating rate (°C/min) | 5°C/min | 9°C/min | 12°C/min |
| Mass temperature (ion source) | 250°C | | |

**Table S2.** Quantitative analysis of 16 VOCs in spray products using two sampling methods.


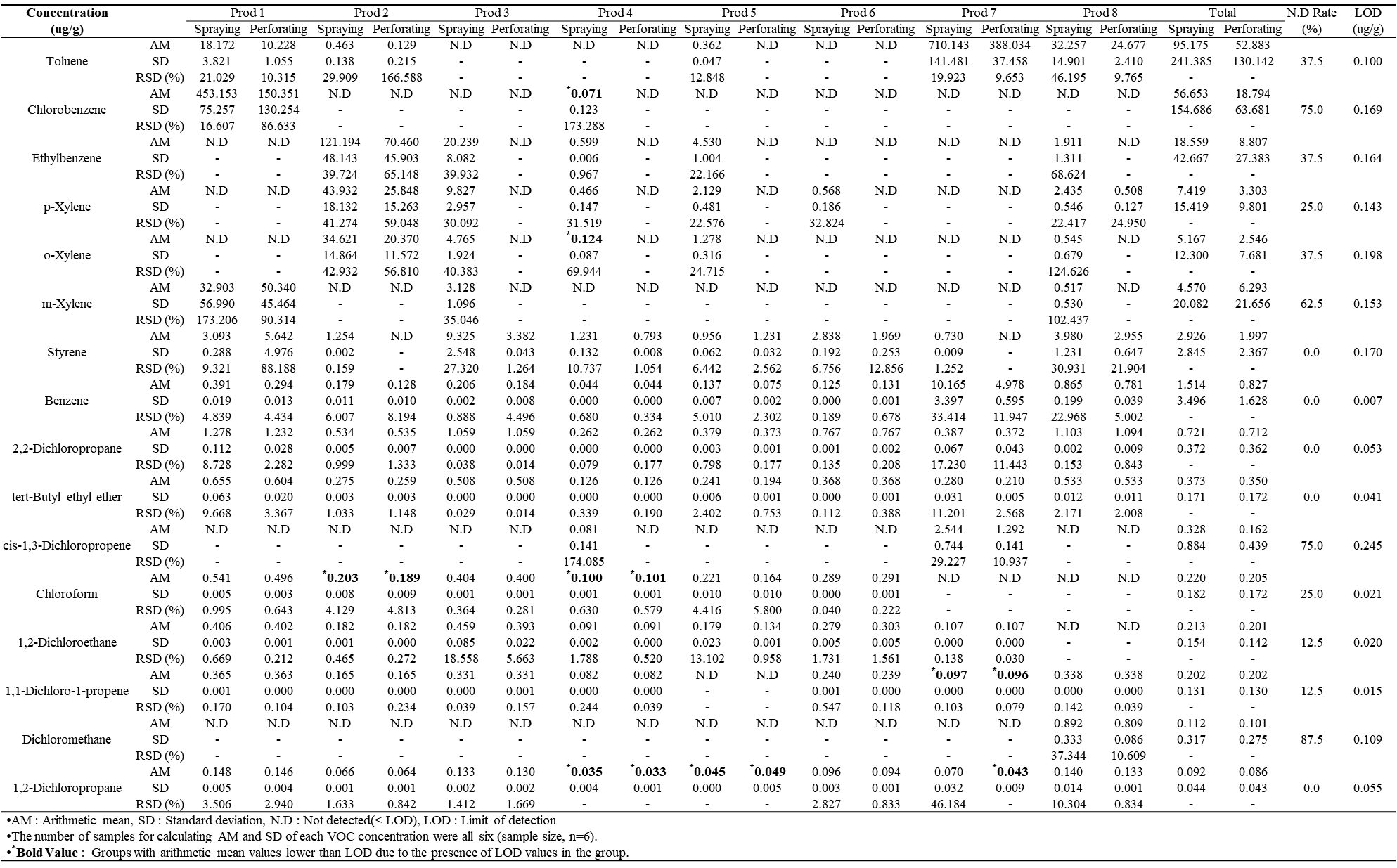


**Table S3.** P-values obtained by statistically comparing the two sampling methods applied to 16 VOCs in consumer aerosol spray products.


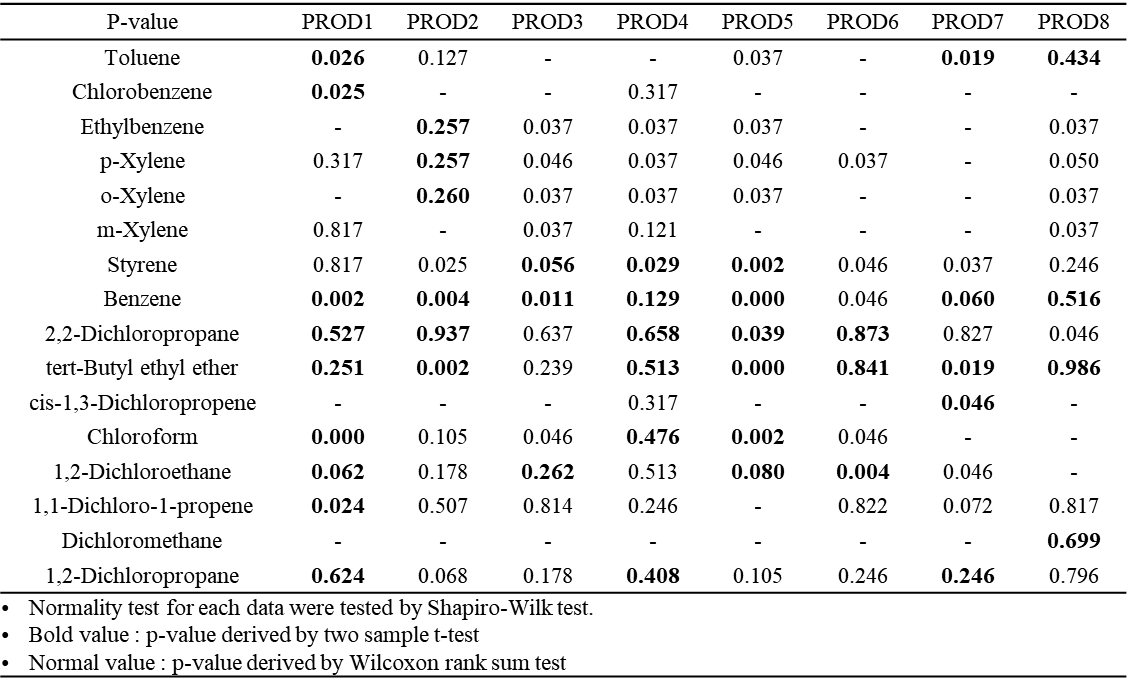


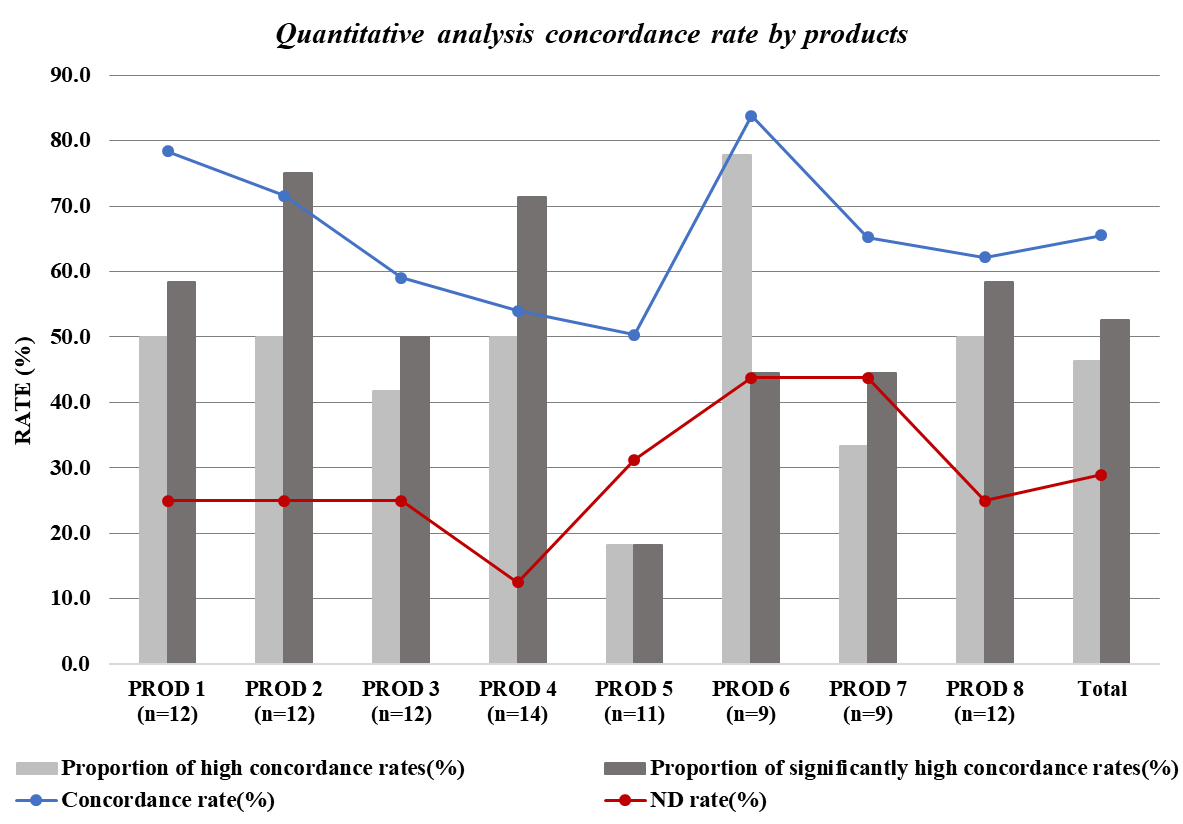
**Figure S1.** Concordance rate between sampling methods by spray product.

Light grey bars: proportion of VOCs with a concordance rate between sampling methods > 90% for the same consumer product.

Dark grey bars: proportion of VOCs for which there was no statistically significant difference (p < 0.05) between sampling methods for the same consumer product.

**Table S4.** Product information and chemical components for each consumer aerosol spray product used in the study.


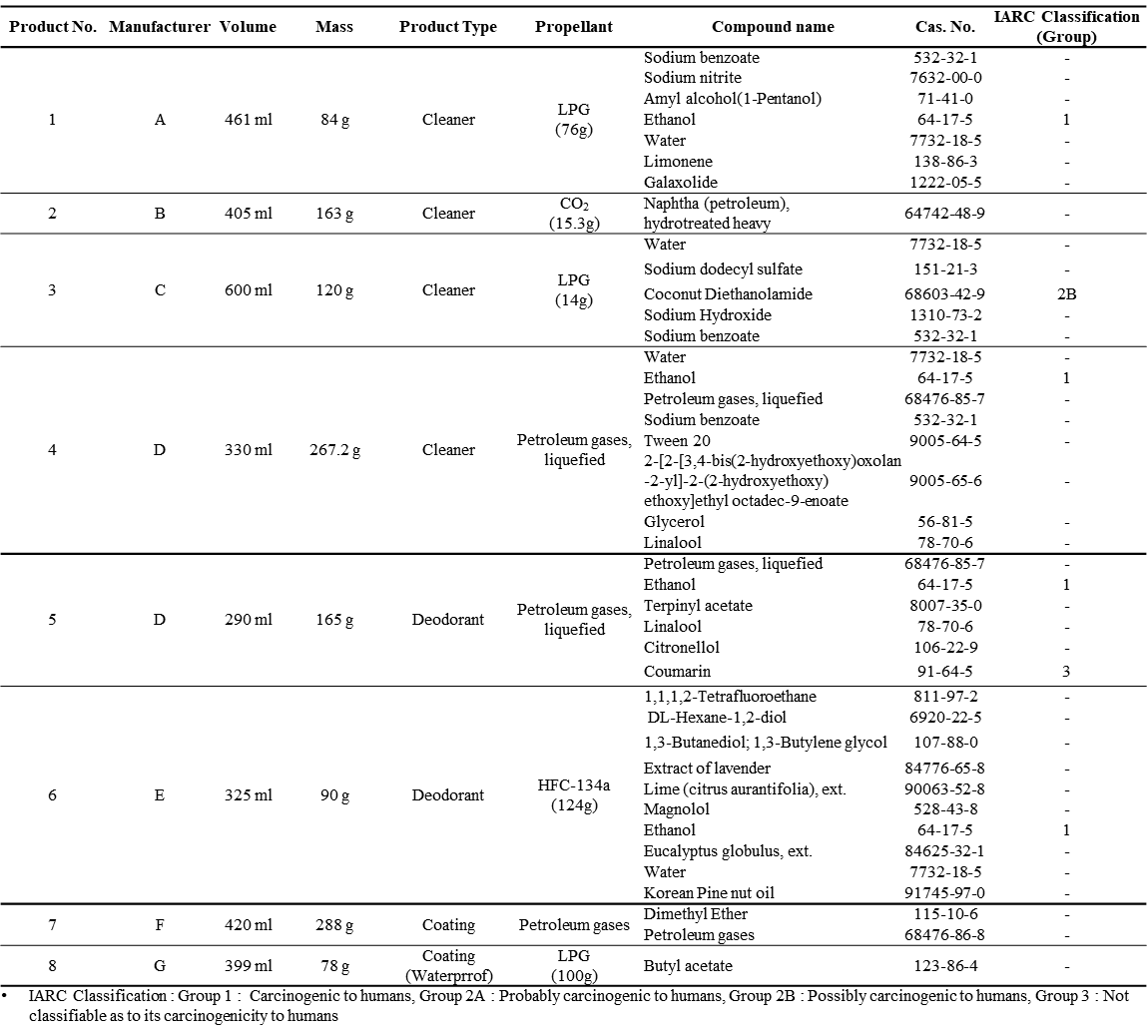


**Table S5.** Chemical properties and detection rates of the VOCs analyzed in the study.


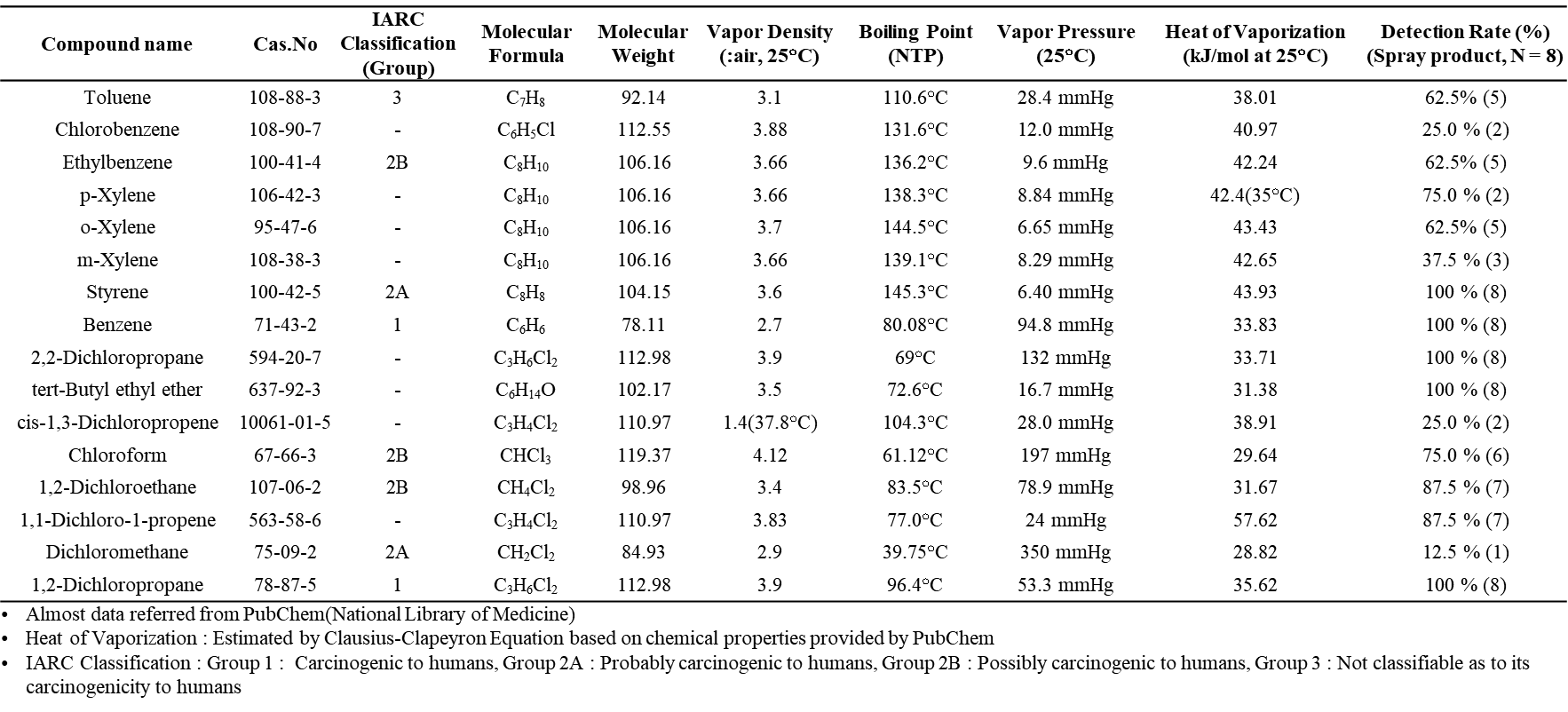


**Table S6.** The concordance rates between sampling methods by product.


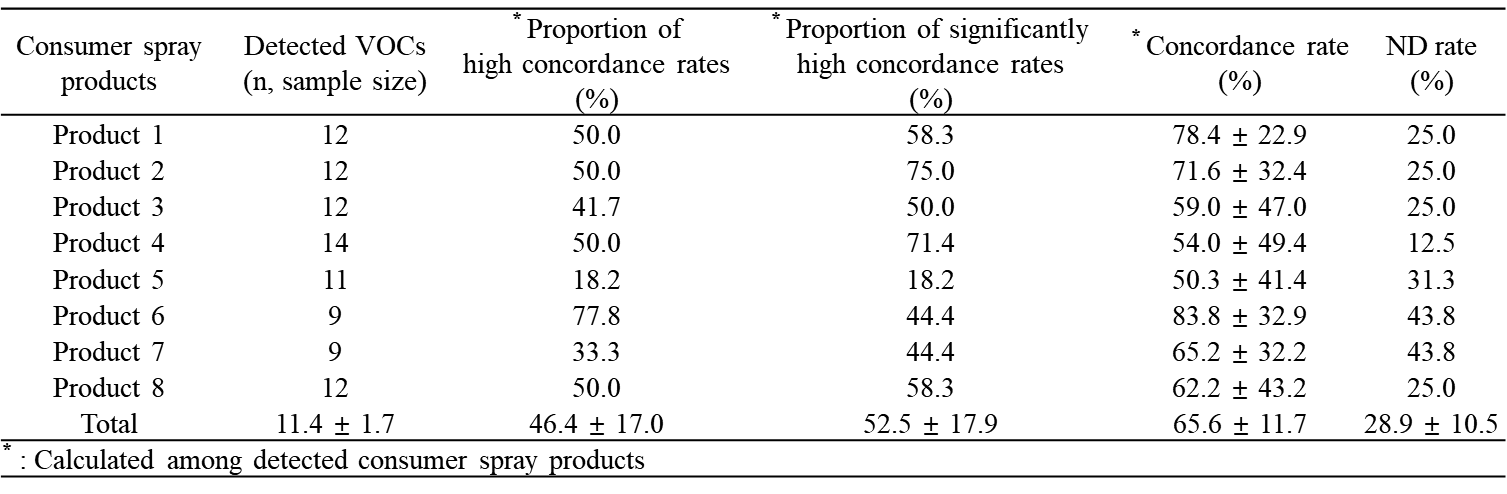


**Table S7.** Quantitative analysis concordance rate according to sampling method by substances.


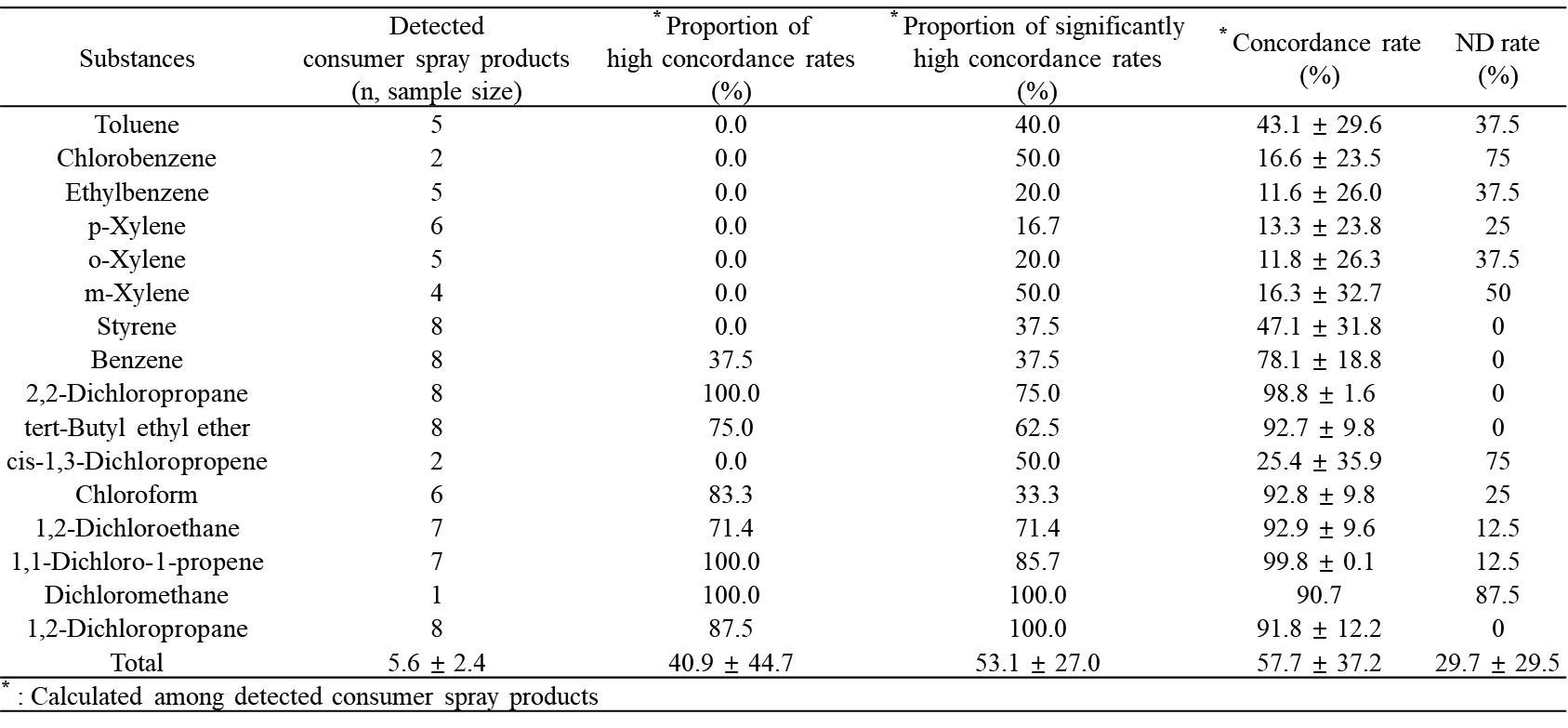


**Table S8.** Relative standard deviation (RSD) of substances among analyzed eight consumer aerosol sprays.


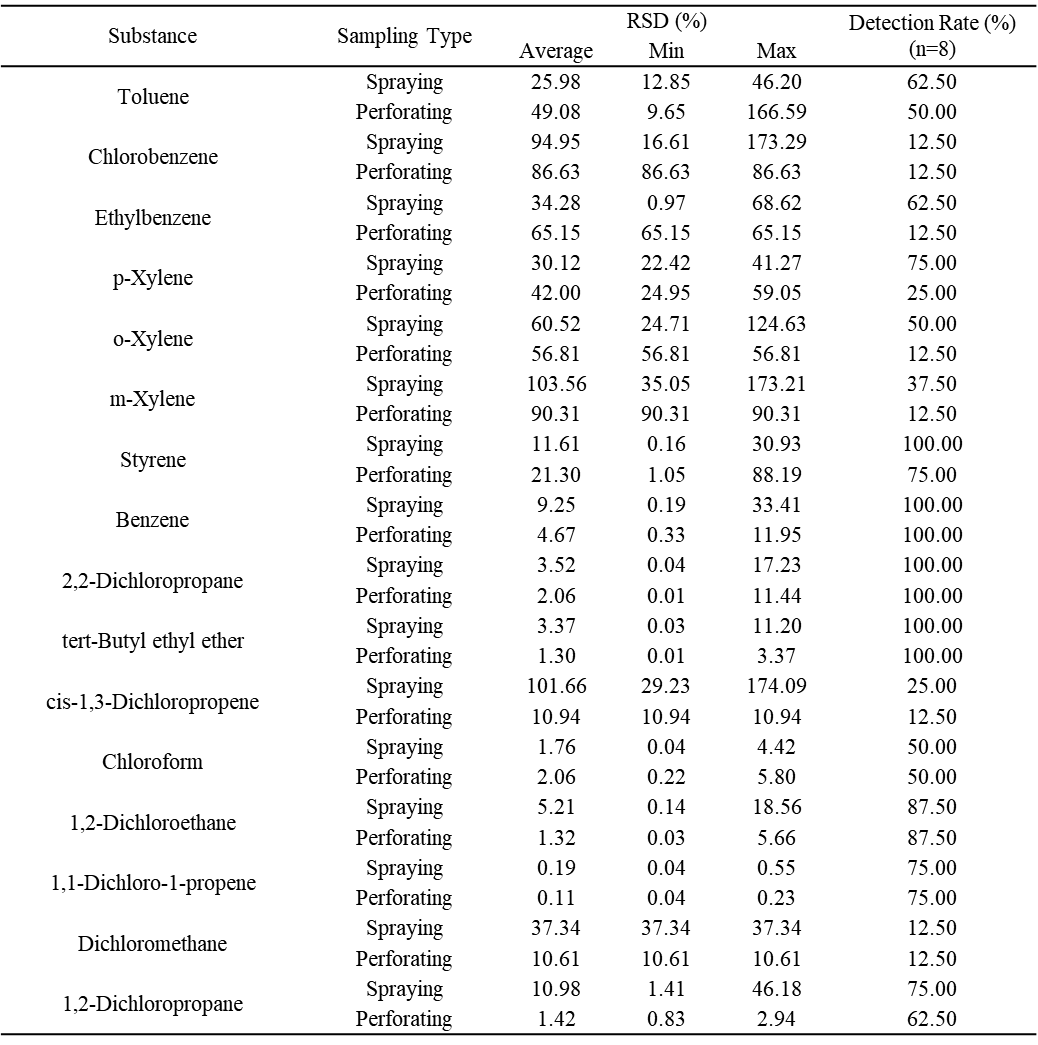


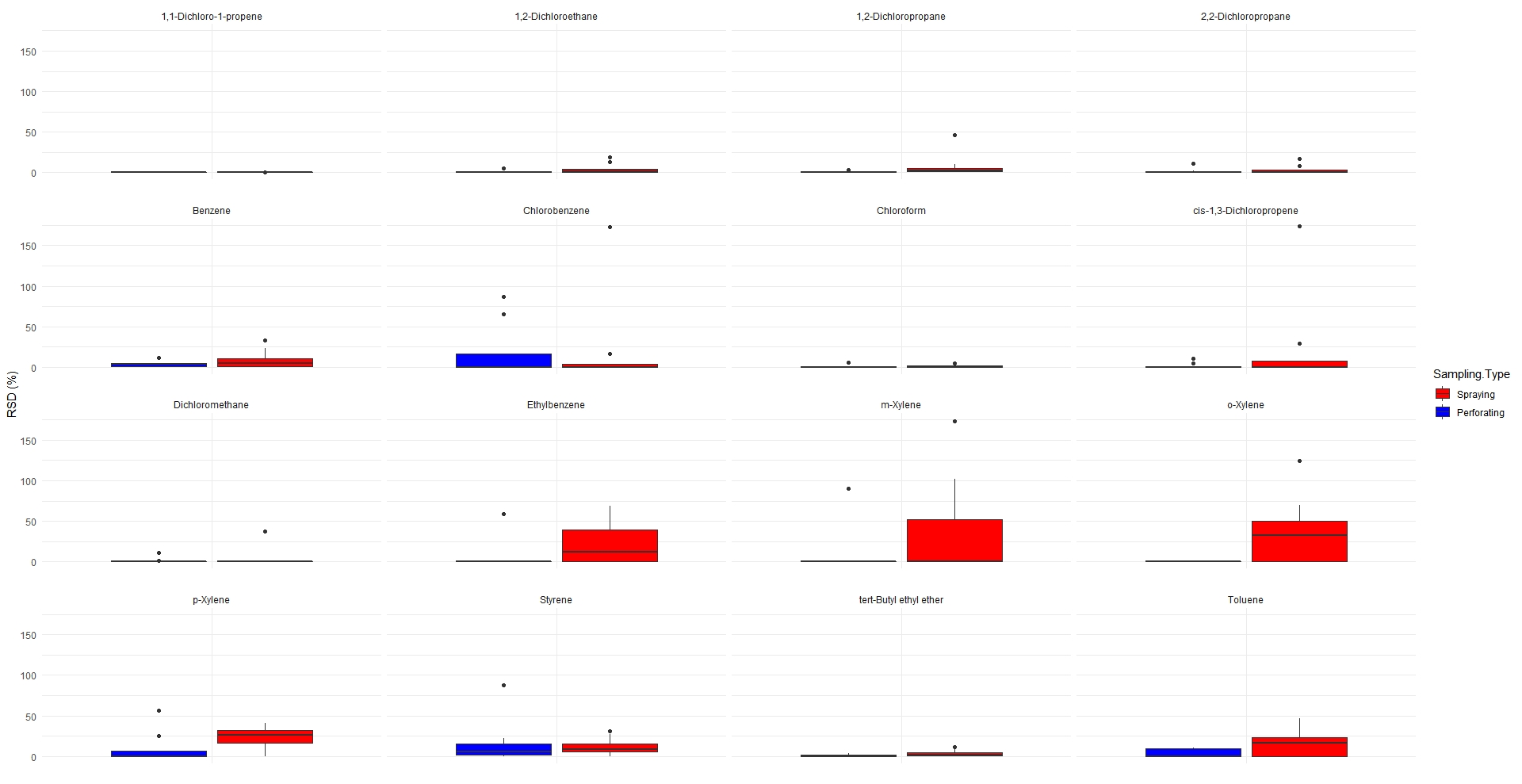


**Figure S2.** Distribution of RSD values in quantitative analysis of eight consumer aerosol sprays for each substance.
